# A Physics–Data Hybrid Framework Using Uncalibrated Consumer CMOS Vision: Pilot Study on Monocular Automatic TUG Assessment Towards Early Parkinson’s Disease Risk Screening

**DOI:** 10.3390/mi17050523

**Published:** 2026-04-25

**Authors:** Yuxiang Qiu, Xiaodong Sun, Fan Yang, Jarred Fastier-Wooller, Shun Muramatsu, Michitaka Yamamoto, Toshihiro Itoh

**Affiliations:** Department of Precision Engineering, Graduate School of Engineering, University of Tokyo, Tokyo 113-8656, Japan

**Keywords:** Timed Up and Go (TUG), gait analysis, calibration-free, monocular vision, phase segmentation, cognitive risk screening, home-based monitoring

## Abstract

The Timed Up and Go (TUG) test is a clinical gold standard for assessing elderly mobility, yet its automated deployment in home-monitoring and resource-limited areas is hindered by high hardware costs and expert calibration requirements. This study introduces a Physics–Data Hybrid framework specifically designed for uncalibrated consumer-grade CMOS cameras, enabling a “plug-and-play” solution for early Parkinson’s disease (PD) risk screening. The proposed pipeline integrates learning-based pose perception with a self-evolving physics model to recover absolute metric-scale motion without manual checkerboard calibration. A noise-adaptive fusion strategy is implemented to reconcile 2D pixel dynamics with 3D kinematic consistency, overcoming the inherent scale ambiguity of monocular vision. Crucially, this framework enables the extraction of high-dimensional spatiotemporal parameters—such as stride length coefficient of variation and mean gait velocity—which provide a finer diagnostic resolution for capturing subtle motor fluctuations than conventional timing-only systems. Results from our pilot study with a cohort of 10 subjects demonstrate that these extracted metric features serve as decisive markers for risk staging simulated by dual-task-induced cognitive-motor-interference, achieving 98% screening accuracy and an overall classification accuracy of 87.32%. This framework provides a robust, low-cost tool for ubiquitous telehealth, potentially supporting early PD risk assessment in underserved populations.

## 1. Introduction

The rapid acceleration of global aging has positioned neurodegenerative diseases, particularly mild cognitive impairment (MCI) and dementia, as critical public health challenges [1]. Early risk screening is paramount for effective intervention, yet clinical diagnosis often lags behind disease onset. Growing evidence suggests that gait is a “window” to cognitive function, as walking requires significant executive resources. Consequently, motor dysfunction often precedes cognitive decline, making dual-task gait performance a robust predictor of dementia risk [2,3]. Among commonly adopted clinical assessments, the Timed Up and Go (TUG) test provides a concise yet comprehensive evaluation of functional mobility by integrating sit-to-stand, walking, turning, and stand-to-sit phases [4].

While the TUG test provides a critical diagnostic window for early PD screening, its effective implementation in home-monitoring or resource-limited remote areas is often hindered by the high cost and operational complexity of professional sensing systems. This creates a significant ‘clinical barrier’ for elderly populations in underserved regions, where early-stage gait abnormalities often go unnoticed due to the lack of accessible screening tools. Traditional manual stopwatch timing [5] is subjective and lacks kinematic detail. While instrumented approaches using wearable sensors [6] or optical motion capture systems (e.g., Vicon) [7] offer high precision, they face a critical trade-off between accuracy and usability. Wearables are often viewed as intrusive by the elderly, leading to poor compliance, whereas motion capture systems are prohibitively expensive and confined to laboratory settings [8]. Meanwhile, advances in microfabrication have enabled miniaturized, low-power MEMS inertial/pressure sensors that underpin many wearable gait-analysis systems, reducing device size and cost [9,10]. Nevertheless, these micromachine-enabled wearables still require proper attachment and calibration to control bias and drift, and thus their real-world deployability in unsupervised home environments remains constrained by user adherence and setup variability [11].

Computer vision-based markerless systems (e.g., Kinect, OpenPose) have emerged as promising alternatives [12]. However, a key practical bottleneck in this field lies in the deployment strategy: most existing high-precision systems rely on rigorous camera calibration (e.g., checkerboard patterns) to solve scale ambiguity [13]. While this ensures accuracy, it renders the system impractical for non-expert users in home environments. Conversely, uncalibrated methods often suffer from pixel jitter and depth ambiguity, leading to unreliable data [14]. A direct alternative to monocular RGB is the use of 3D sensing hardware such as structured-light or time-of-flight RGB-D cameras, which provide metric depth without explicit checkerboard calibration. However, these systems introduce additional opto-electronic complexity and remain susceptible to distance-dependent depth noise and temporal fluctuations under real-world conditions, including surface reflectance, ambient illumination, and multipath interference, potentially reintroducing depth jitter into derived gait parameters [14,15,16,17]. Technically compact 3D ranging modules leveraging micro-optics and MEMS components, such as beam-steering micromirrors in solid-state LiDAR, are becoming more feasible, underscoring the ongoing trade-off between hardware complexity and deployability in home monitoring scenarios [18]. Furthermore, regarding diagnostic algorithms, there is a divergence between “black-box” deep learning models, which require massive datasets, and interpretable biomechanical models. For clinical staging of cognitive load, specifically distinguishing between mild and high loads, purely data-driven methods often struggle with individual heterogeneity and lack physiological interpretability [19]. Therefore, a practical home-deployable TUG system must simultaneously (i) avoid calibration, (ii) output metric-scale kinematics with low jitter, and (iii) provide interpretable staging under limited data.

Existing monocular vision studies often focus on temporal segmentation (timing) but struggle with scale ambiguity, which filters out high-dimensional spatiotemporal metrics, which serves as critical biomarkers such as stride length variability. To address all these challenges, this study leverage widely accessible consumer-grade sensors (embedded CMOS optics) to develop a plug-and-play, low-cost hybrid framework for automated non-contact TUG analysis and cognitive screening. We propose a methodology that synergizes data-driven deep learning perception with physics-based optical modeling to achieve automatic motion segmentation, gait parameter extraction, and cognitive load screening.

Specifically, intelligent algorithms are first employed to extract 2D keypoints and 3D skeletal representations from video streams. Subsequently, to circumvent the reliance on camera intrinsic parameters which are often unknown and variable across consumer devices—we leverage accessible external geometric constraints (i.e., camera and subject height) based on pinhole imaging principles. This approach enables precise distance estimation using a single uncalibrated monocular camera. Furthermore, by introducing a multi-modal fusion technique that integrates the 2D pixel-derived distance with adaptive 3D skeletal pose data, we effectively suppress pixel jitter inherent in sensor noise and pose estimation uncertainty. Finally, a hierarchical classification system driven by Lasso regularization is then established to screen and stage cognitive impairment. It presents a pilot study to validate the technical feasibility and metrological accuracy of this proposed uncalibrated pipeline.

The main contributions of this paper are summarized as follows:

1. A physics–data hybrid framework: A unified pipeline integrating pose estimation with geometric constraints for fully automated TUG-phase segmentation and parameter extraction from uncalibrated video.
2. Uncalibrated metric distance estimation: A self-calibration method that substitutes unknown intrinsics with practical geometric priors for consumer-grade deployment.
3. Robust Jitter Suppression: A noise-adaptive fusion strategy that stabilizes metric trajectories by reconciling 2D pixel dynamics with 3D kinematic consistency.
4. Explainable Diagnosis as a Pilot Study: An L1-regularized hierarchical model that provides interpretable feature-level evidence for staging, supporting clinically meaningful screening beyond a single aggregate metric and validating the metrological accuracy.

## 2. Framework and Methods

### 2.1. Framework Architecture

**Figure 1:**
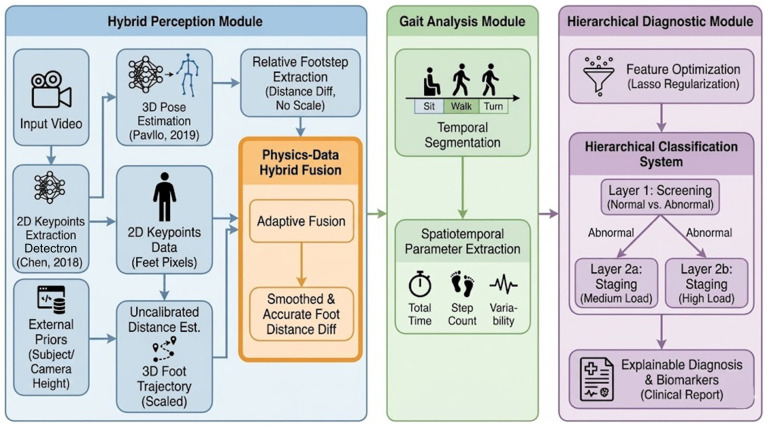
Overall pipeline of proposed framework [20,21].

The proposed framework addresses the challenge of quantifying the TUG test using a non-calibrated monocular camera. As illustrated in Figure 1, the processing pipeline consists of three cascaded modules: (1) Perception module, which extracts key points and estimates pose from raw video sequence, self-calibrate and recovers the metric scale via an optimization-based solver, and integrates geometric constraints with deep learning-based depth priors to mitigate perspective-induced jitter; (2) Gait Analysis module: automated Phase Segmentation, and Parameter Extraction, which detects clinical events and computes spatiotemporal gait metrics; and (3) Hierarchical diagnostic module to screen and stage cognitive load via hierarchical Logistic regression.

### 2.2. Pose Estimation and Coordinate Synchronization

Let the input video sequence be denoted as $V={\left\{I_{t}\right\}}_{t=1}^{T}$, where $I_{t}$ is the t-th frame and T is the total number of frames. For each frame t, we employ a top-down pose estimator [20] to extract the 2D pixel coordinates of J=17 anatomical keypoints:

(1) $$P_{uv}(t)\in R^{J\times 2},$$

where the j-th keypoint is represented as $\left(u_{j}(t),v_{j}(t)\right)$ in pixel coordinates. Simultaneously, a monocular 3D pose estimation network VideoPose [21] predicts the corresponding relative (scale-ambiguous) 3D keypoints:

(2) $$P_{dl}(t)\in R^{J\times 3},$$

where the j-th keypoint is $\left(x_{j}(t),y_{j}(t),z_{j}(t)\right)$ in the camera-centered coordinate system. To ensure temporal consistency, a low-pass filter (e.g., Butterworth) is applied to suppress high-frequency acquisition noise.

### 2.3. Optimization-Based Geometric Self-Calibration

**Figure 2:**
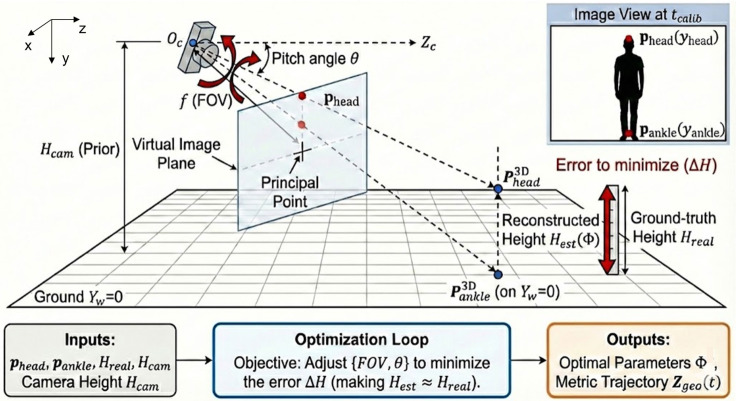
Schematic illustration of the optimization-based geometric self-calibration.

A critical limitation of monocular video is the lack of metric depth information, resulting in inherent scale ambiguity. To recover metric scale without external calibration patterns, we propose an optimization-based geometric self-calibration strategy using the subject’s known body height and camera’s height, as weak constraints. The geometric configuration and the back-projection are illustrated in Figure 2.

#### 2.3.1. Calibration Frame Selection

The system first identifies an optimal calibration frame $t_{calib}$ where the subject exhibits a fully upright posture. This is achieved by maximizing the vertical pixel projection of the body:

(3) $$t_{calib}=arg\;max\;t\left(y_{ankle}(t)-y_{head}(t)\right),$$

where $y_{ankle}(t)$ and $y_{head}(t)$ denote the vertical pixel coordinates of the ankle midpoint and head keypoint, respectively.

#### 2.3.2. Parameter Solver

We model the camera using a pinhole model characterized by an unknown field of view (FOV) and pitch angle (θ) (Figure 2). Let Φ={FOV,θ} denote the camera parameter set, and $H_{real}$ be the ground-truth height of the subject. The calibration is formulated as a non-linear least squares optimization:

(4) $$\hat{\Phi }=arg\;min\;\Phi \;{∥\;M(P_{uv}(t_{calib});\Phi )-H_{real}\;∥}^{2},$$

Here, M(·) denotes the geometric back-projection function that maps 2D pixel coordinates to a 3D metric reconstruction under the ground-plane constraint Y=0. The optimization is solved using the Trust Region Reflective algorithm, yielding the optimal parameter set $\hat{\Phi }$. Based on $\hat{\Phi }$, a metric geometric depth trajectory $Z_{geo}(t)$ is generated for the entire sequence.

### 2.4. Noise-Adaptive Multi-Modal Fusion

While $Z_{geo}(t)$ provides metric depth estimates, it is susceptible to perspective jitter, particularly when the subject is distant from the camera. In contrast, the deep learning-based depth prior $Z_{dl}(t)$ is temporally smoother but lacks metric scale. To leverage the complementary strengths of both modalities, we introduce a noise-adaptive fusion mechanism.

#### 2.4.1. Jitter Quantification

We define a real-time noise indicator η(t) by measuring the high-frequency residual of the geometric depth. Let ${\tilde{Z}}_{geo}(t)$ be the smoothed baseline of $Z_{geo}(t)$. The instantaneous noise level is computed within a temporal window of size W frames:

(5) $$\eta (t)=\frac{1}{W}\sum_{k=t-W/2}^{t+W/2}|\;Z_{geo}(k)-{\tilde{Z}}_{geo}(k)\;|,$$

#### 2.4.2. Dynamic Weighting and Fusion

A dynamic confidence weight w(t)∈[0,1] is determined by a piecewise linear transfer function:

(6) $$w(t)=\{\begin{array}{cc} 1.0, & \eta (t)\leq \tau_{stable}\\ \frac{\tau_{jitter}-\eta (t)}{\tau_{jitter}-\tau_{stable}}, & \tau_{stable}<\eta (t)<\tau_{jitter}\\ 0.1, & \eta (t)\geq \tau_{jitter} \end{array},$$

where $\tau_{stable}$ and $\tau_{jitter}$ are empirically selected thresholds corresponding to stable and unstable geometric conditions, respectively. The lower bound 0.1 preserves weak metric anchoring even under severe jitter.

The final fused metric depth $Z_{final}(t)$ is computed as:

(7) $$Z_{final}(t)=w(t)\cdot Z_{geo}(t)+(1-w(t))\cdot s\cdot Z_{dl}(t),$$

where s is a scale alignment factor derived from the standard deviation ratio of the two signals:

(8) $$s=\frac{std(Z_{geo})}{std(Z_{dl})},$$

This fusion strategy prioritizes geometry-based depth in stable conditions while smoothly transitioning to the deep-learning prior during high-noise intervals.

### 2.5. Kinematic Phase Segmentation

**Figure 3:**
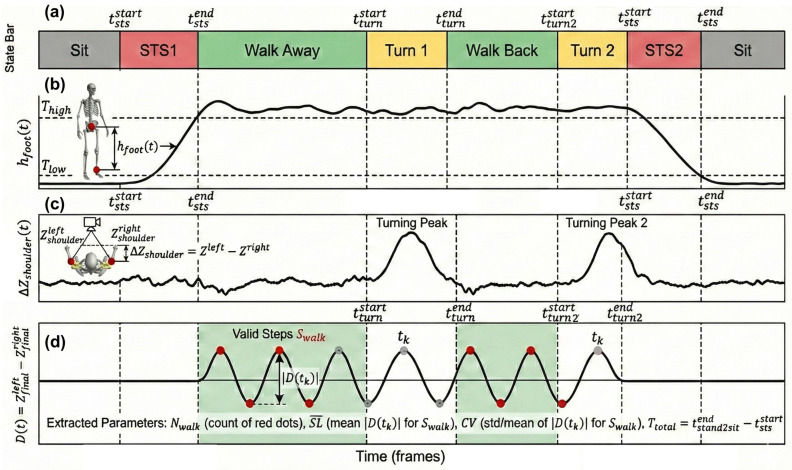
Conceptual Schematic illustration of signal-driven segmentation and feature extraction over a complete TUG session. (**a**) TUG-phase state; (**b**) vertical displacement for STS transitions’ detection: STS1 and STS2 represent sit_to_stand and stand_to_sit, respectively; (**c**) shoulder depth difference for turning’s detection; (**d**) foot depth difference for step detection.

Traditional velocity-based segmentation is prone to false positives under noisy measurements. Therefore, we implement a robust state machine based on vertical displacement and torso rotation (Figure 3).

- Sit-to-Stand/Stand-to-Sit: As shown in Figure 3b, we monitor the vertical distance $h_{foot}(t)$ between the ankle midpoint and the root joint (e.g., pelvis). Subject-specific thresholds $T_{high}$ and $T_{low}$ are determined from the static range of motion. The *Sit-to-Stand* phase ends when $h_{foot}(t)\geq T_{high}$, whereas the *Stand-to-Sit* phase begins when $h_{foot}(t)\leq T_{low}$.
- Turning: As shown in Figure 3c, the turning phase is detected by the depth difference between the left and right shoulders:
  (9) $$\Delta Z_{shoulder}(t)=|Z_{L\_sh}(t)-Z_{R\_sh}(t)|,$$

A significant peak in $\Delta Z_{shoulder}(t)$ indicates torso rotation relative to the camera plane.

### 2.6. Automatic Parameter Extraction

After phase segmentation, the system automatically extracts a compact state-space feature vector $x\in R^{d}$ that summarizes the spatiotemporal performance of a TUG session. Following the implementation in our pipeline, the extracted parameters include total test duration, step statistics, turning and sit-to-stand dynamics, and gait variability.

#### 2.6.1. Step Event Detection

Let the fused depth-difference signal between the left and right feet be denoted as:

(10) $$D(t)=Z_{final}^{left}(t)-Z_{final}^{right}(t),$$

where $Z_{final}^{left}(t)$ and $Z_{final}^{right}(t)$ are the fused metric depths (Section 2.4) of the left and right foot keypoints at frame t, respectively.

Step events are detected as prominent peaks and valleys in D(t), as shown in Figure 3d. Let $S_{all}={\left\{t_{k}\right\}}_{k=1}^{N_{total}}$ denote the set of all detected step candidates (including both peaks and valleys), where $N_{total}$ is the total count of step candidates within the entire session. To avoid false detections outside walking phases, we further restrict valid steps to those falling within the walking-away and walking-back intervals:

(11) $$S_{walk}=\{t_{k}\in S_{all}|t_{k}\in \mathrm{Walk}\_\mathrm{Away}\cup \mathrm{Walk}\_\mathrm{Back}\},$$

where Walk_Away and Walk_Back are determined from the segmented state machine. Let $N_{walk}=|S_{walk}\;|$ denote the number of valid walking steps.

#### 2.6.2. Stride-Length Proxy and Variability

For each valid step event $t_{k}\in S_{walk}$, we define a stride-length proxy:

(12) $$SL_{k}=|D(t_{k})|,$$

The mean stride length is then computed as:

(13) $$\bar{SL}=\frac{1}{N_{walk}}\sum_{k=1}^{N_{walk}}S\;L_{k},$$

and the stride-length variability is quantified using the coefficient of variation:

(14) $$CV=\frac{\sigma_{SL}}{\mu_{SL}}\times 100\%,$$

where $\mu_{SL}$ and $\sigma_{SL}$ are the mean and standard deviation of ${\left\{SL_{k}\right\}}_{k=1}^{N_{walk}}$, respectively.

#### 2.6.3. Temporal TUG Parameters

Let fps denote the frame rate. The total TUG duration is defined as:

(15) $$T_{total}=\frac{t_{stand,end}^{sit}-t_{sit,start}^{stand}}{fps},$$

where $t_{stand,\mathrm{e}nd}^{sit}$ and $t_{sit,start}^{stand}$ are the detected boundary indices of the Sit-to-Stand and Stand-to-Sit transitions.

The turning duration is computed as:

(16) $$T_{turn}=\frac{t_{\mathrm{Turn}\_1,\mathrm{end}}-t_{\mathrm{Turn}\_1,\mathrm{start}}}{fps},$$

and the sit-to-stand duration is:

(17) $$T_{sts}=\frac{t_{sit,end}^{stand}-t_{sit,start}^{stand}}{fps},$$

These parameters are explicitly included in the final reported state-space metrics.

#### 2.6.4. Step Counts and Walking Speed

We define the total number of steps:

(18) $$N_{total}=|S_{all}|,N_{walk}=|S_{walk}|,$$

Finally, an estimated walking speed is computed by:

(19) $$V_{walk}=\frac{\bar{SL}\cdot N_{walk}}{T_{total}},$$

which corresponds to the accumulated walking distance divided by the full TUG duration.

#### 2.6.5. State-Space Representation

The extracted features form a compact state vector:

(20) $$x={\left[T_{total},\;V_{walk},\;T_{turn},\;T_{sts},\;N_{total},\;N_{walk},\;\bar{SL},\;CV\right]}^{⊤},$$

where $T_{total}$ is the total test duration, $N_{total}$ and $N_{walk}$ denote the total/valid walking step counts, $\bar{SL}$ and CV are the mean stride-length proxy and its coefficient of variation, $T_{turn}$ and $T_{sts}$ indicate turning and sit-to-stand durations, and $V_{walk}$ is the estimated walking speed. These features are automatically computed from the fused depth trajectories and the segmented phases, producing a structured clinical report for each TUG session.

### 2.7. PD Risk Stratification via Hierarchical Sparse Logistic Regression

Sparse logistic regression with L1 regularization (LASSO) is a widely used baseline for gait-related clinical screening due to its interpretability and robustness under limited sample sizes, and it is commonly evaluated using leave-one-out cross-validation (LOOCV) [22]. Building on the TUG state-space features extracted in Section 2.6, we perform a three-class stratification into Normal/Healthy (N), Mild cognitive burden (M) (early PD-like condition), and Severe cognitive burden (P) (PD-equivalent condition). To improve robustness under small datasets, we adopt a two-stage hierarchical scheme instead of a flat three-class classifier.

In Stage 1, we screen N versus abnormal A={M,P} using: $x^{\left(1\right)}={\left[T_{total},\;V_{walk},\;T_{turn},\;N_{total},\;CV\right]}^{⊤},$ which matches the implemented screening feature set. In Stage 2, samples predicted as abnormal are graded into M versus P using: $x^{\left(2\right)}={\left[V_{walk},\;N_{walk},\;\bar{SL},\;T_{total},\;T_{turn},\;T_{sts},\;CV\right]}^{⊤},$ consistent with the implemented grading feature set.

All features are z-score-standardized and evaluated with LOOCV. Stage 1 selects the regularization strength via internal validation, while Stage 2 uses a fixed setting for stable grading. The final prediction $\hat{y}\in \{N,M,P\}$ is obtained by cascading the two stages: trials classified as normal remain N; otherwise, Stage 2 assigns M or P.

This hierarchical design preserves interpretability while reducing the complexity of direct three-class learning under small-sample conditions. By using an interpretable sparse classifier on clinically meaningful TUG-derived state-space features, the proposed framework enables low-cost, contactless, and scalable in-home screening for early Parkinsonian risk. In this case, the whole pipeline could be summarized in Algorithm 1.
**Algorithm 1.** Physics–Data Hybrid Automatic TUG Assessment Algorithm**Input:** video $V=\{I_{t}{\}}_{t=1}^{T}$, subject height $H_{real}$ **Output:** TUG state vector x and class $\hat{y}\in \{N,M,P\}$**Pose estimation:** obtain $P_{uv}(t)$ and $P_{dl}(t)$; apply temporal smoothing.**Self-calibration:** select $t_{calib}$ and solve $\hat{\Phi }=\{FOV,\theta \}$ using height constraint $H_{real}$.**Depth fusion:** compute $Z_{geo}(t)$, $Z_{dl}(t)$; estimate η(t)→w(t); output fused $Z_{final}(t)$.**Phase segmentation:** detect Sit-to-Stand, Walk-Away/Back, Turning, Stand-to-Sit.**Metric extraction:** compute $T_{total},V_{walk},T_{turn},T_{sts},N_{total},N_{walk},\bar{SL},CV$ and form x.**Hierarchical classification:**○Stage 1: classify N vs. A={M,P} using $x^{\left(1\right)}$.○Stage 2: if abnormal, classify M vs. P using $x^{\left(2\right)}$.○output $\hat{y}\in \{N,M,P\}$.


## 3. Experiment and Results

### 3.1. Experimental Setups and Data Acquisition

#### 3.1.1. Data Acquisition Environment and Participants

To evaluate the robustness of the proposed framework across diverse real-world scenarios, experiments were conducted in two distinct indoor environments: a standard conference room and a typical living room. As detailed in Table 1, the experimental setup varied in terms of camera installation heights (1.1 m and 0.9 m) and subject starting positions relative to the camera, creating a dataset with high geometric variability. To establish a rigorous spatial ground-truth, we utilized a laser level for axis alignment and a high-precision steel tape to mark physical reference intervals on the floor. These markers provided absolute coordinate benchmarks to verify the accuracy of the spatiotemporal gait metrics extracted by the monocular system.

Video data were acquired using a consumer-grade smartphone (iPhone 16) mounted on a tripod. The sampling rate was fixed at 30 fps, with a resolution of 1920 × 1080 pixels, ensuring sufficient temporal resolution for gait analysis. A pilot cohort of ten healthy volunteers participated in this preliminary technical validation. Their demographic information, including age and height, is summarized in Table 2. For the linguistic background, the native language of subjects is Mandarin or Japanese, while the foreign language is either Japanese or English.

#### 3.1.2. The TUG Protocol and Phase Definition

**Figure 4:**
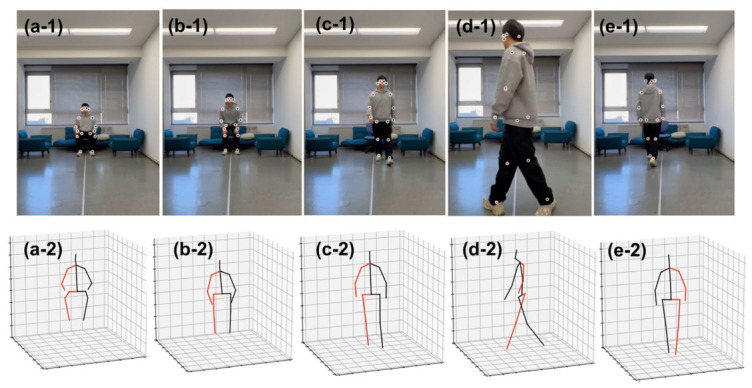
Experiment process in the living room with 3D motion extraction. (**a-1**,**a-2**) Static Sitting; (**b-1**,**b-2**) Sit-to-Stand; (**c-1**,**c-2**) Walk-Towards; (**d-1**,**d-2**) Turn-around; (**e-1**,**e-2**) Walk-Away. In each figure, 1 (1st row) represents slices in raw video signal, and 2 (2nd row) represents estimated 3D motions.

Each participant performed the standard Timed Up and Go (TUG) test following the sequence illustrated in Figure 4. The complete motion cycle comprises: (1) Static Sitting, (2) Sit-to-Stand Transition, (3) Walking towards the Camera (approx. 3 m), (4) Turning (180°), (5) Walking Away from the Camera, (6) Turn-and-Sit, and (7) Static Sitting. This strict phase definition allows for the precise segmentation of kinematic events. The Far_point represents farthest place at the sitting place (Figure 4(a-1,a-2)), while the Near_Point denotes the nearest place at the first turning point (Figure 4(d-1,d-2)).

#### 3.1.3. Dual-Task Paradigm for Cognitive Load Simulation

The adoption of the Dual-Task (DT) paradigm with healthy participants, rather than confirmed Parkinson’s Disease (PD) patients, is strategically grounded in both clinical utility and methodological feasibility. While advanced PD presents overt motor symptoms easily detectable via visual inspection, the primary clinical challenge lies in prodromal screening, where gait abnormalities remain sub-clinical and nearly invisible. Validating algorithms with actual prodromal cohorts is often impractical for pilot studies due to the necessity of multi-year longitudinal tracking to confirm retrospective diagnoses.

To simulate the motor impairments observed in different stages of PD, specifically the loss of gait automaticity, we employed a DT Paradigm to induce Cognitive-Motor Interference (CMI) [23,24]. The experiment was divided into three varying levels of cognitive load:

1. Normal Class (N): Participants performed the TUG test naturally without any secondary task, establishing a baseline for normal gait performance.
2. Mild Cognitive Load (M): Participants performed the TUG test while simultaneously engaging in a Serial Sevens Subtraction task (starting from 1000), vocalizing the results in their native language (L1). This condition simulates mild cognitive distraction.
3. Parkinsonian-like proxy conditions (P): Participants performed the Serial Sevens task but were required to vocalize results in their foreign language (L2). This configuration imposes a significantly higher executive burden on the prefrontal cortex, aiming to simulate the severe gait freezing or variability often seen in advanced PD or high-stress scenarios [25].

### 3.2. Self-Calibrated Distance Measurement

#### 3.2.1. Qualitative Trajectory Reconstruction

**Figure 5:**
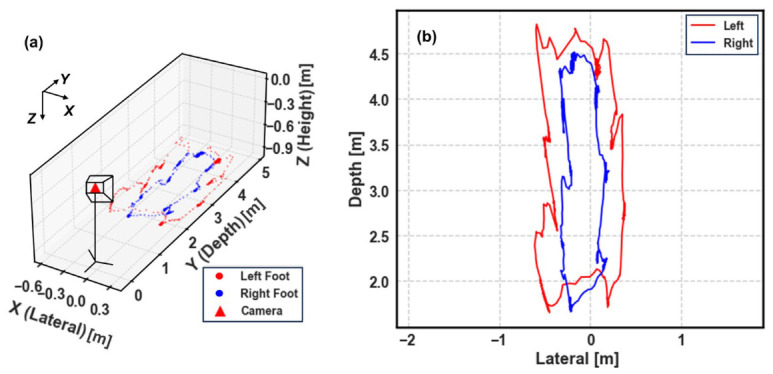
Visualization of the reconstructed 3D foot trajectories for a complete TUG session. (**a**) A 3D perspective view illustrating the spatial relationship between the uncalibrated camera (red triangle) and the subject’s movement. (**b**) A top-down (bird’s-eye) projection on the lateral-depth plane.

To intuitively validate the self-calibration algorithm, we first reconstructed the participant’s motion trajectory in metric space. Figure 5 visualizes the real-time kinematic tracking of a representative TUG session (Subject 1, Living room).

Figure 5a provides a 3D isometric view of the session relative to the camera coordinate system. Despite the lack of external calibration patterns, the system successfully recovers a coherent 3D structure where the walking path extends approximately 3 m in depth—consistent with the standard TUG protocol (approx. 3 m). Based on these points, the approximate location of human could be estimated.

Furthermore, the top-down projection in Figure 5b reveals the geometric quality of the reconstruction. The trajectory exhibits a clear U-shaped pattern, with straight walking segments and a well-defined turning curve. Crucially, the lateral separation between the left (red) and right (blue) feet is preserved without the ‘flatness’ artifact often seen in purely monocular estimations. In addition, the left and right foot are well recognized even after the subject turns around, which contributes to accurate depth difference estimation in the following pipeline. This visualization confirms that our physics-based geometric constraint effectively solves the scale ambiguity, transforming normalized pixel coordinates into a physically meaningful metric space suitable for clinical gait analysis.

#### 3.2.2. Quantitative Error Analysis

In the absence of frame-by-frame ground truth from motion capture systems in the remote setting, we validated the measurement accuracy by leveraging the standardized geometric constraints of the TUG protocol. Specifically, we assessed the system’s metric fidelity through three distinct spatial indicators: the Near Point Error, the Far Point Error, and the resultant Walking Distance Error.

**Figure 6:**
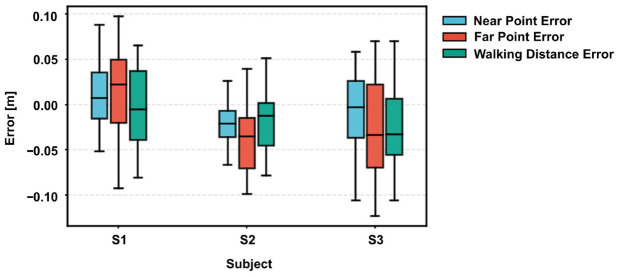
Typical Absolute Error Distributions.

As illustrated in Figure 6, the system demonstrated robust metrological performance across all subjects. Quantitatively, the measurement errors for all three indicators were tightly confined, with the inter-quartile range consistently remaining within less than 0.1 m. It is worth noting that the system exhibited high robustness against individual variations. Despite the differences in height and gait patterns among subjects S1, S2, and S3, no significant performance degradation was observed. While minor systematic biases were present—such as a slight positive bias for S1 and a negative bias for S2—the Walking Distance Error (Green bar), which is the most critical parameter for clinical gait speed calculation, remained stable and accurate across the board.

Beyond the overall accuracy, a closer inspection of the error distribution reveals an inherent physical characteristic of monocular vision. As evident in Figure 6, the variance of the Far Point Error (in Red) is noticeably larger than that of the Near Point Error (in Blue). This phenomenon is not an algorithmic artifact but is attributable to the optical principles of the pinhole camera model, where the depth resolution decays quadratically with the distance. Consequently, a single-pixel jitter in the keypoint detection translates to a larger metric fluctuation at far point than at near point. However, crucially, our results demonstrate that the Walking Distance Error does not escalate proportionally. This suggests that the proposed Noise-Adaptive Fusion algorithm effectively smooths out the high-frequency fluctuations in the far field, ensuring that the derived stride parameters remain reliable for clinical assessment.

### 3.3. Performance of Kinematic Phase Segmentation and Parameter Extraction

**Figure 7:**
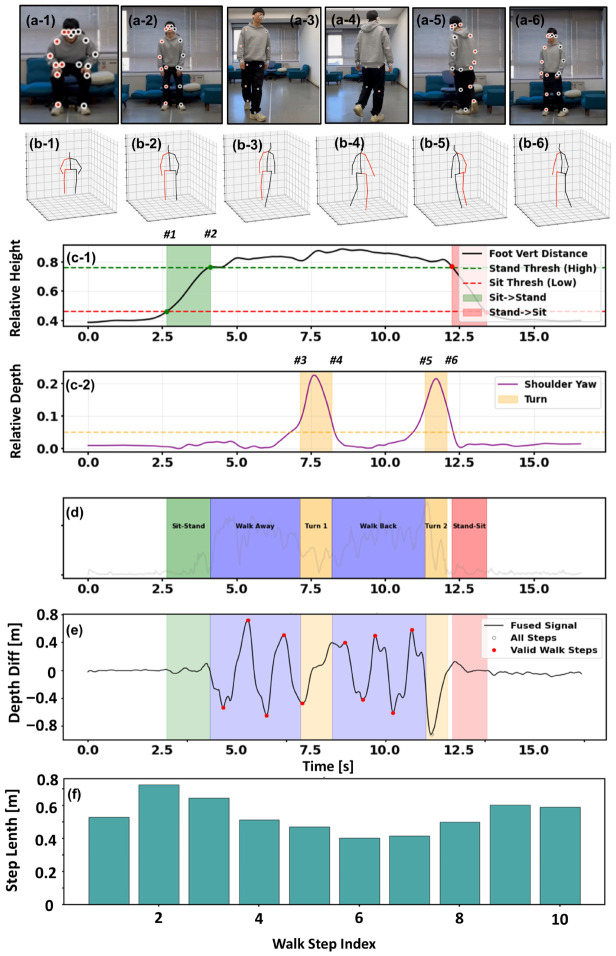
Qualitative visualization of the signal-driven kinematic phase segmentation and automated parameter extraction pipeline on a representative TUG test sample. (**a-1**–**a-6**,**b-1**–**b-6**) Keyframes from the input video and corresponding reconstructed 3D skeletons at distinct functional moments. (**c**) Signal-based event detection: (**c-1**) Vertical displacement identifies the Sit-to-Stand (STS) and Stand-to-Sit phases based on subject-specific height thresholds; (**c-2**) Shoulder depth difference captures torso rotation, distinctively marking the two turning phases (Turn 1 & Turn 2). (**d**) The resulting comprehensive temporal segmentation mask covering the complete TUG cycle. (**e**) Step event detection on the fused foot depth difference signa, where valid walking steps (red dots) are isolated from non-walking artifacts (hollow circles) within the segmented walking intervals. (**f**) The final extracted stride lengths for each validated step, illustrating the system’s capability to capture spatial gait variability. The numbered markers (#1–#6) indicate the key temporal events during the TUG test: #1 and #2 represent the start and completion of the Sit-to-Stand transition; #3 and #4 delineate the start and end of the mid-course turn; #5 and #6 mark the beginning and conclusion of the turn event prior to Stand-to-Sit phase.

While the spatial accuracy discussed in the previous section establishes the system’s metrological reliability, the clinical essence of the TUG test resides in its temporal structure—specifically, how a patient transitions between sitting, walking, and turning. Figure 7 provides a holistic view of this automated analysis pipeline, illustrating the transformation of raw motion data into granular clinical insights.

#### 3.3.1. Automated Phase Segmentation

The first challenge in automated TUG analysis is decomposing the continuous video stream into semantically meaningful phases. Our system addresses this by monitoring specific kinematic signatures that mirror the physical milestones of the test.

Initially, the system focuses on the vertical dynamics to identify postural transitions. As visualized in Figure 7(c-1), the Foot Vertical Distance signal remains flat while the subject is seated but exhibits a sharp, logistic-like ascent as the subject rises. By applying adaptive thresholds (indicated by the green and red dashed lines), the algorithm delineates the Sit-to-Stand (green zone with markers #1–#2 in Figure 7(c-1)) and Stand-to-Sit (red zone) phases. This signal-level detection aligns perfectly with the ground-truth video frames (Figure 7(a-1,a-2)) and the corresponding skeletal reconstructions (Figure 7(b-1,b-2)), confirming that the start and end of the test are captured with frame-level precision.

Subsequently, the analysis shifts to the horizontal plane to detect turning motions. The Shoulder Yaw signal, shown in Figure 7(c-2), serves as a sensitive indicator for rotational movement. The signal is characterized by distinct bell-shaped peaks (highlighted in yellow) that correspond to the subject’s turning actions. By isolating these peaks, the system effectively separates the straight-line walking phases from the Turn events (markers #3-#4 and #5-#6 in Figure 7(c-2)). The integration of these vertical and rotational cues results in the fully segmented timeline shown in Figure 7d, replacing manual stopwatch timing with an objective chronological breakdown.

#### 3.3.2. Gait Event Detection and Filtering

Once the “Walk Away” and “Walk Back” phases are temporally isolated (the blue regions in Figure 7d), the system zooms in to analyze the micro-structure of the gait.

The algorithm reconstructs the depth trajectory of the ankles (Figure 7e), revealing the rhythmic, quasi-sinusoidal pattern inherent to human walking. Crucially, the system is designed to distinguish between effective walking progression and non-gait adjustments. As evident in the signal, steps that contribute to locomotion are marked as “Valid Walk Steps” (red dots). Conversely, the small, shuffling adjustments typically observed during turning or pre-seating are correctly classified as transitional movements (gray circles). This selective filtering ensures that the subsequent parameter calculation is derived strictly from steady-state walking, preventing the “noise” of functional transitions from skewing the clinical metrics.

#### 3.3.3. Spatiotemporal Parameter Quantification

Ultimately, these validated kinematic events are translated into quantitative spatial metrics. Figure 7f presents the step-by-step length analysis for the entire session, offering clinical insights that go beyond a simple average.

The bar chart reveals the natural variability in the subject’s gait strategy. For instance, a clear trend of deceleration is observable: the subject initiates with longer strides, but significantly shortens their stride length as they approach the turn. This step-to-step variability, captured automatically by the pipeline, provides a valuable window into the subject’s motor planning and dynamic stability. By visualizing and analyzing the individual variance, the system delivers a comprehensive “Stopwatch-Free” assessment.

#### 3.3.4. Construction of the Clinical State Vector

To condense the complex spatiotemporal dynamics into a computable format for cognitive load screening, we aggregated the extracted features into a unified Gait State Vector. This vector serves as a “digital phenotype” of the subject’s mobility, encapsulating global performance, rhythm stability, and sub-task proficiency.

As summarized in Table 3, the state vector is constructed by concatenating seven key physiological parameters derived from the segmented phases, as shown in $x=[T_{total},\;V_{walk},\;T_{turn},\;T_{sts},\;N_{total},\;N_{walk},\;\bar{SL},\;CV{]}^{⊤}$. This structured representation allows for a granular analysis of the subject’s biomechanical status. By decomposing the complex TUG sequence into quantifiable components, we analyze the performance across the following three critical dimensions:

1. Global and Kinematic Metrics: The vector includes the Total Time, which remains the gold standard for TUG scoring. Complementing this, the Step Counts and Average Stride Length provide context on the stride strategy used to achieve that time. For instance, a subject might achieve a normal time by compensating for short strides with a higher cadence, a nuance captured only by this multi-dimensional vector.
2. Dynamic Stability and Variability: A critical component of our vector is the Stride Coefficient of Variation, calculated as the ratio of the standard deviation to the mean step length. In the reported trial, the relatively high variability quantitatively reflects the deceleration strategy observed in Figure 7f, where stride length decreased significantly during the maneuvering phases. This metric is widely regarded as a sensitive biomarker for neurological control [26].
3. Functional Sub-Task Proficiency: Finally, the vector isolates specific functional capabilities through Turn Duration and Sit-to-Stand Duration. These independent metrics allow clinicians to pinpoint whether a mobility deficit stems from dynamic balance (turning) or lower limb strength (rising), rather than relying solely on the aggregate test duration.

**Table 3:** Component of state vector.

Parameter	Label	Unit	Value
Total Time	$T_{total}$	s	12.2
Step Count (Total)	$\;N_{total}$	1	14
Step Count (Walk)	$\;N_{walk}$	1	12
Avg Stride	$\bar{SL}$	m	0.53
Stride CV	CV	%	21.65
Turn Duration	$T_{turn}$	s	1.2
Sit-Stand Duration	$\;T_{sts}$	s	1.6

### 3.4. Diagnostic Classification Effectiveness

To translate the multidimensional Gait State Vector into an actionable clinical decision, we evaluated its discriminative power using a sparse logistic regression with L1 regularization. The objective was to categorize subjects into three clinically distinct groups: N, M, and P. As summarized in Table 4, the system achieved an overall classification accuracy of 87.32%. However, the true clinical value of the system is best understood by analyzing the specific distribution of these results across two dimensions: healthy screening and severity grading.

#### 3.4.1. Robustness in Healthy Screening

**Figure 8:**
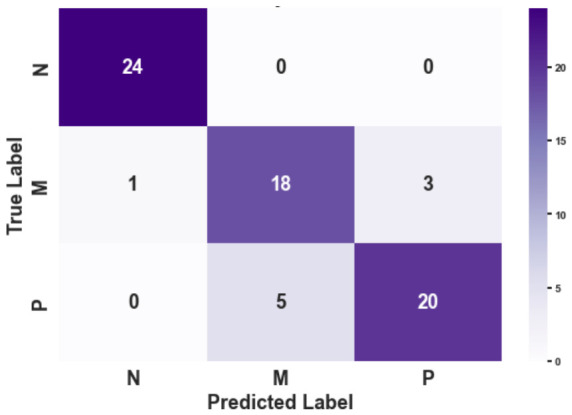
Confusion Matrix on Diagnostic Classification.

The confusion matrix (Figure 8) and associated metrics (Table 4) provide a realistic assessment of the system’s screening performance. In a clinical screening context, the Recall (Sensitivity) for pathological conditions is the most critical metric to minimize the risk of undiagnosed cases.

Notably, the Precision of 0.96 for the ‘Normal’ (N) class reveals that one ‘Mild’ case was misclassified as ‘Normal.’ This instance of missed diagnosis (False Negative) underscores the inherent challenge in distinguishing sub-clinical gait changes from healthy variations. Such overlap typically occurs at the boundary of cognitive-motor interference, where the subtle decrease in gait automaticity in some subjects may not yet exceed the statistical threshold of the physics–data hybrid model.

Despite these boundary challenges, the framework demonstrates high overall diagnostic efficacy, achieving an aggregate classification accuracy of 87.32%, as detailed in Table 4. While the current pilot study on 10 subjects highlights a small margin of leakage for ‘Mild’ cases, the framework maintains sufficient sensitivity to flag the vast majority of at-risk gait patterns. These pilot findings suggest the potential robustness of the physics–data hybrid approach in transforming uncalibrated monocular video into actionable health metrics for remote screening.

#### 3.4.2. Resolution of Pathological Severity

In contrast to the absolute separation of healthy subjects, the differentiation within the pathological spectrum—specifically between Mild (M) and Severe (P)—presented a more nuanced challenge. As shown in the off-diagonal elements of Figure 8, misclassifications were strictly confined between these two adjacent stages (e.g., 5 ‘P’ subjects classified as ‘M’). Consequently, the F1-scores for the M and P groups dipped slightly to 0.80 and 0.83, respectively.

This overlapping pattern is, however, physiologically understandable. The progression from mild motor impairment to early-stage Parkinson’s is a continuous phenotypic spectrum rather than a discrete step change. The “confusion” captured by our classifier reflects the inherent ambiguity of these transitional stages. Importantly, the confusion matrix reveals a strictly hierarchical error pattern: pathological subjects were confused with each other, but never with the healthy control group. This confirms that while the system’s sensitivity to the exact grade of severity may be influenced by biological variability, its sensitivity to the presence of motor pathology remains robust.

## 4. Discussion

### 4.1. Evaluation of Noise-Adaptive Fusion Strategy with Ablation Test

**Figure 9:**
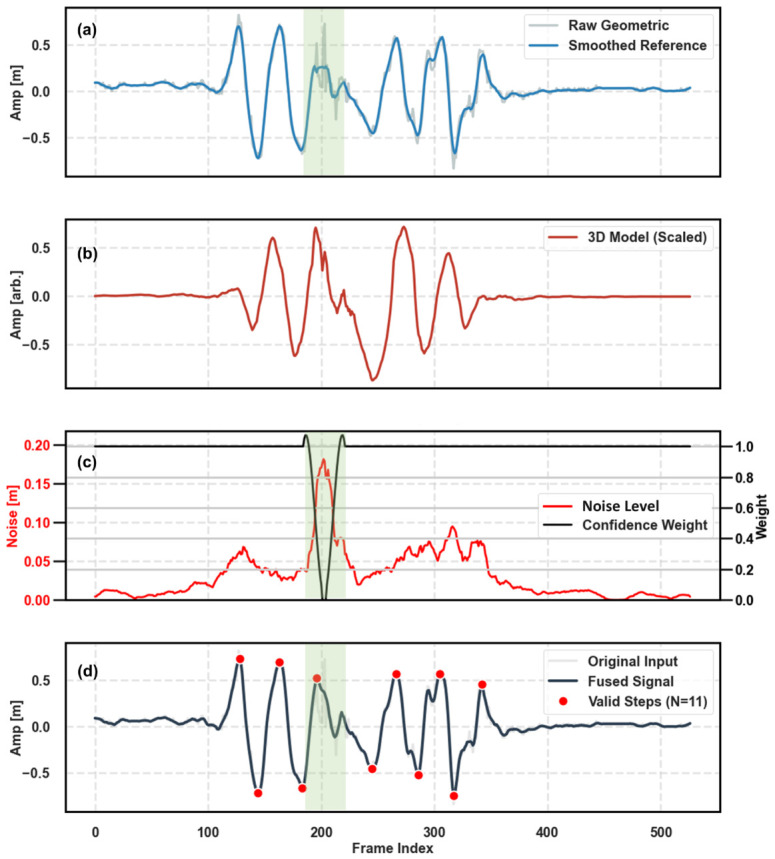
Visual ablation study illustrating the efficacy of the noise-adaptive fusion strategy. (**a**) The raw geometric distance estimation (gray) exhibits severe high-frequency pixel jitter, particularly within the noise-prone interval (green shaded region), deviating from the smoothed reference. (**b**) The 3D skeletal relative trajectory serves as a robust shape reference with high smoothness, albeit lacking metric scale (arbitrary units). (**c**) The dynamic weight generation mechanism: a surge in the detected Noise Level (red) triggers a sharp decrease in the Confidence Weight (black) assigned to the geometric signal. (**d**) The final fused signal (dark blue) successfully suppresses the jitter by adaptively prioritizing the 3D skeletal consistency in low-confidence regions while preserving the metric scale of the geometric prior, enabling accurate step detection (red dots).

To evaluate the contribution of the multi-modal fusion module, we conducted a qualitative ablation study, as shown in Figure 9. Figure 9a presents the baseline performance of the uncalibrated geometric estimation (Raw Geometric). While it captures the general trend, it is susceptible to significant high-frequency fluctuations—termed ‘pixel jitter’—caused by keypoint detection instability (highlighted in the green shaded region). Relying solely on this signal would lead to false step detections and inaccurate stride length calculation.

In contrast, the 3D skeletal trajectory derived from the deep learning model (Figure 9b) demonstrates superior smoothness and kinematic consistency but lacks physical scale information (arbitrary units).

Our adaptive fusion strategy bridges this gap by dynamically arbitrating between these two sources. As visualized in Figure 9c, the system continuously monitors the signal quality. When the localized noise level (red curve) spikes within the jitter-prone region, the algorithm automatically penalizes the geometric signal by reducing its confidence weight (black curve) to near zero. Consequently, the final fused signal (Figure 9d) effectively ‘in-paints’ the noisy segment using the smooth morphology of the 3D skeleton while retaining the metric scale provided by the geometric prior. This mechanism significantly enhances the signal-to-noise ratio, ensuring robust step event detection (red dots) even in the presence of sensor instability.

### 4.2. Diagnostic Efficacy as a Screening Tool

**Figure 10:**
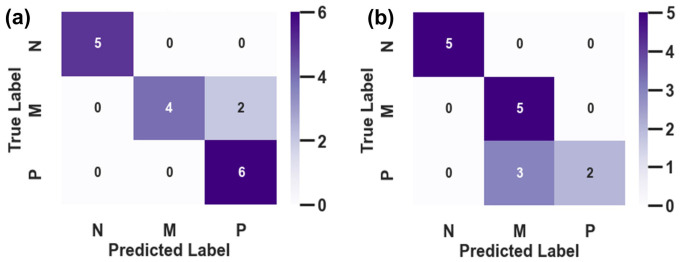
Confusion matrices illustrating the diagnostic performance of the hierarchical classification model on two representative validation sets. The classes correspond to Normal (N), Mild Cognitive Load (M), and High Cognitive Load (P). (**a**) Results for Subject 1, showing high sensitivity for the high-load class (P) with minor misclassification of mild-load cases. (**b**) Results for Subject 2, demonstrating perfect precision for the normal and mild classes, with a tendency towards conservative prediction in high-load cases. Note that in both scenarios, the Normal class (N) is perfectly isolated from the pathological states, validating the robustness of the first-stage screening.

To further evaluate the clinical reliability of our hierarchical framework, Figure 10 presents the confusion matrices derived from two representative validation scenarios (Subject 1 and Subject 2). The diagonal dominance in both matrices underscores the overall effectiveness of the extracted kinematic features.

A critical observation is the perfect separation of the Normal (N) class across both subjects. As shown in the first row and column of Figure 10a,b, there were no false positives or false negatives involving the healthy control group. This confirms that the first layer of our hierarchical model (Screening: Normal vs. Abnormal) is highly robust, satisfying the primary requirement for a home-based screening tool—minimizing missed diagnoses.

Regarding the second layer (Staging: Mild vs. High Load), the model exhibits interpretable variations reflecting individual heterogeneity. In Figure 10a (Subject 1), the model achieved 100% recall for the High Load (P) class, though two Mild Load (M) instances were over-estimated as High Load. Conversely, Figure 10b (Subject 2) shows a more conservative trend, where three High Load instances were misclassified as Mild Load, yet the Normal class itself was identified with 100% precision. These minor misclassifications between adjacent stages (M and P) are clinically acceptable and expected, likely attributed to the subtle kinematic differences between moderate and severe cognitive loads. Nevertheless, the system effectively distinguishes ‘Normal’ from ‘Impaired’ with high confidence.

### 4.3. Clinical Value Beyond Total Time

Total Time provides a strong coarse screening cue in within-subject settings, but it is sensitive to subject-dependent baselines and therefore insufficient for fine-grained grading under cross-subject evaluation. Consistent with this observation, the subject-wise distributions of Total Time show clear baseline shifts, which can cause overlap between the Mild (M) and severe (P) conditions.

By augmenting Total Time with interpretable phase- and variability-related descriptors (e.g., stride Length Coefficient of Variation (CV), sit-to-stand duration, turning duration, and walking step statistics), the proposed state-space representation improves grading robustness and offers clinically actionable insights on how the performance deteriorates, rather than only how long the test takes. The L1-regularized model further mitigates overfitting by selecting a compact feature subset under small-sample conditions, supporting an interpretable and deployable screening pipeline.

**Figure 11:**
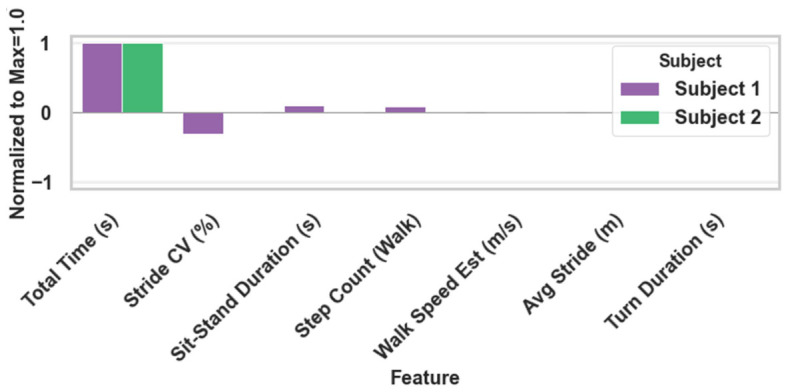
Regularization sweep across multiple features demonstrating the trade-off between model sparsity and classification accuracy.

As shown in Figure 11, we identified an optimal operating point where the model achieves peak cross-validation accuracy while selecting a minimal, highly compact subset of stable features (e.g., stride length variability and turning duration). This strategic feature selection not only ensures metrological robustness against small-sample variance but also explicitly highlights the most clinically actionable descriptors, thereby supporting an interpretable and deployable screening pipeline.

In addition, the prominent retention of Stride CV by the L1-regularized model is clinically profound. In neurodegenerative assessments, Stride CV serves as a sensitive biomarker for the loss of gait automaticity, a recognized hallmark of prodromal Parkinson’s disease. This data-driven prioritization of Stride CV aligns with clinical findings, demonstrating that recovering scale-dependent variability is essential for achieving higher screening sensitivity than traditional timing-only metrics.

**Figure 12:**
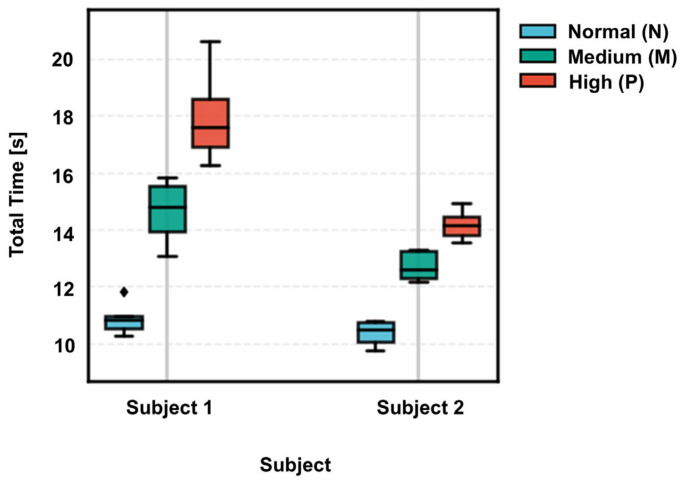
Distribution of total time across subjects.

Figure 12 shows substantial inter-subject baseline shifts in Total Time, indicating that absolute completion time is strongly subject-dependent. While Total Time can separate conditions within an individual, cross-subject distributions overlap, especially for intermediate severity, which limits global-threshold or single-feature classification. Therefore, Total Time is best suited for coarse screening, whereas robust staging requires complementary phase- and variability-related descriptors.

### 4.4. Limitations and Future Work

As a pilot study, the current validation was conducted on a limited cohort (n = 10) to verify the algorithmic feasibility of the physics–data hybrid framework. While the system demonstrated high sensitivity to cognitive loads within this group, the sample size is insufficient to derive generalized clinical cut-off scores or calculate statistical power. However, this study successfully establishes the proof-of-concept that uncalibrated video can recover metric-scale TUG parameters, laying the technical foundation for future large-scale clinical trials involving diverse pathological populations.

Despite the system’s efficacy, three limitations inherent to monocular vision remain. First, environmental sensitivity: Performance relies on image quality; low lighting or severe occlusions (e.g., loose clothing, furniture) can disrupt pose estimation, though interpolation mitigates minor data loss. Second, standardized protocol dependency: The geometric self-calibration relies on a planar floor and standardized movement (e.g., 3 m path and 180-degree turns). While these constrain the system’s use in unconstrained terrains, they intentionally align with the mandatory TUG clinical protocol to ensure diagnostic validity. Third, single-subject constraint: While static background clutter is filtered, dynamic interference from pets or family members in uncontrolled homes challenges the current tracking logic.

Future work will address these by integrating temporal constraints and advanced algorithms (e.g., DeepSORT [27]) to handle multi-person tracking and dynamic occlusions, migrating pipeline to edge computing (e.g., smartphones) to ensure raw video is processed locally, and by expanding to longitudinal studies with diverse pathological cohorts to validate the predictive value of the gait state vector. Furthermore, the precision of gait metrics in unconstrained environments is often challenged by non-stationary and heavy-tailed measurement noise, stemming from rapid movements or environmental interference. While our noise-adaptive fusion provides basic stabilization, incorporating advanced robust estimation frameworks—such as the robust Kalman filter based on the Normal-Bernoulli distribution [28]—could further enhance methodological depth. Such frameworks are particularly effective in modeling non-Gaussian outliers, providing a more rigorous interpretation of reliability when processing heterogeneous visual data.

### 4.5. Comparison with Related Works

Recent markerless, video-based TUG and gait assessment studies can be broadly categorized into two technical routes: (i) 3D-enabled sensing (depth/RGB-D cameras) and (ii) monocular RGB pipelines that infer motion from 2D/3D pose representations. Table 5 summarizes representative works and highlights the key trade-offs that motivate our design.

Depth/RGB-D-based systems provide metric depth measurements without requiring explicit checkerboard calibration, enabling reliable timing and phase-level analysis under relatively controlled installation conditions. For example, Kinect-style depth sensing has been used to automate the TUG test and extract kinematic descriptors with strong practicality for lab-to-clinic translation [5]. More recently, temporal deep models operating on RGB-D skeleton sequences have further improved subtask segmentation performance and reinforced the value of phase-level descriptors beyond coarse timing measures [29]. Nevertheless, depth/RGB-D solutions inherently introduce additional hardware and deployment constraints (device availability, mounting position, privacy/acceptability, and maintenance), which can limit large-scale “plug-and-play” adoption in unconstrained home environments [5,30].

In contrast, monocular RGB approaches are attractive for home deployment because they require only a conventional camera, but they face two persistent bottlenecks: scale ambiguity and temporal instability (jitter). Some studies achieve gait parameter extraction by using multi-view recordings and semi-automatic steps (e.g., guided marking) to stabilize keypoint trajectories and recover more reliable geometry, which improves validity but increases operational complexity and reduces accessibility for non-expert users [31]. Other monocular pipelines focus on subtask recognition or temporal segmentation via video-based activity classification, offering robust phase labeling but often not targeting stable metric 3D trajectories that are directly comparable across sessions or subjects [32]. Pose-based monocular TUG automation further demonstrates the feasibility of full video pipelines, yet still inherits the fundamental limitations of monocular reconstruction—scale-ambiguous 3D estimates and depth/pose jitter that can propagate into gait parameters, especially at longer ranges [30].

Our framework is positioned to bridge these two routes. As shown in Table 5, we retain the deployment advantage of a single uncalibrated monocular camera, while explicitly addressing monocular bottlenecks through optimization-based self-calibration for metric scale recovery and a noise-adaptive fusion mechanism that suppresses perspective-induced jitter. Compared with depth-camera solutions [5,30], our approach avoids specialized 3D hardware while still outputting metric-scale kinematics suitable for quantitative parameter extraction. Compared with monocular pipelines relying on semi-automatic steps [31] or segmentation-first designs without explicit metric recovery [32], our method provides a principled path to stable metric trajectories. Finally, relative to prior pose-based monocular TUG automation [29], our explicit scale anchoring and jitter-aware fusion improve the reliability of phase segmentation and enable a compact, interpretable TUG state vector for screening and staging under limited data.

**Table 5:** Comparison with related works based on marker-less vision sensors.

Work	Sensor/Setup	Marker-Less Representation	Metric Scale/Calibration	Automation Level	Main Outputs/Tasks	Typical Limitations
[5]	Depth camera (Kinect)	3D pose	Metric depth/No checkerboard needed	Automated	Phase segmentation + enriched kinematics + falling risk evaluation	Require RGB-D/depth hardware; deployment and placement constraints.
[31]	Two-view RGB	2D pose keypoint trajectories	Metric via multi-view geometry and guided heel marking	Semi-automated	Spatiotemporal parameter extraction	Relies on multi-view geometry and manual assistance
[32]	Single-view videos	2D pose keypoint trajectories	Scale ambiguous	Automated	Phase segmentation	No distance-based metrics (e.g., stride CV)
[30]	Monocular RGB	2D keypoints+ 3D pose (scale-ambiguous)	Scale ambiguous	Automated	Phase segmentation	No distance-based metrics (e.g., stride CV) + temporal jitter reduce metric reliability in unconstrained home setups
[29]	RGB-D (Kinect)	RGB-D skeleton/pose sequence + Dilated TCN	Metric depth/No checkerboard needed	Automated	Phase segmentation	Require RGB-D hardware + trained model parameters
This work	Uncalibrated monocular RGB	2D keypoints + 3D pose (scale-ambiguous) + self-calibrated geometric depth + noise-adaptive fusion	Metric scale recovered + stabilized 3D trajectories/No checkerboard needed	Automated	Phase segmentation + spatiotemporal parameter extraction + interpretable PD risk screening/staging	Require further clinical validation on diagnosed cohorts

## 5. Conclusions

This study presented a robust, plug-and-play framework for automated TUG assessment and cognitive impairment screening using uncalibrated monocular vision. By integrating a geometric 3D human model with a novel 2D pixel distribution fusion technique, we effectively overcame the two primary barriers to home-based monitoring: the complexity of camera calibration and the interference of environmental jitter in scale recovery.

Our preliminary experimental results demonstrate that the proposed hierarchical classification system achieves high diagnostic precision, with 98% accuracy in screening (Baseline vs. cognitive-load conditions) and 87.32% accuracy in staging cognitive load severity. A key clinical finding of this work is the nuanced role of gait features: while Total Time serves as a robust universal biomarker for cognitive decline, it is insufficient on its own to capture the heterogeneity of patient responses. Our feature importance analysis revealed that different individuals adopt distinct compensatory strategies (e.g., conservative deceleration vs. systemic gait deterioration) under cognitive load. Therefore, the inclusion of multi-dimensional spatiotemporal parameters (e.g., stride variability, sit-to-stand duration), optimized via Lasso regularization, is critical for enhancing model sensitivity and preventing overfitting in small-sample cohorts.

In summary, this physics–data hybrid approach offers a scalable, non-intrusive solution for early PD’s screening. It demonstrates that automated, multi-parametric gait analysis holds significant potential to reduce misdiagnosis rates compared to traditional manual timing, paving the way for ubiquitous home health monitoring.

Principal conclusions of this work indicate that: (1) The proposed uncalibrated framework demonstrates high robustness and competitive performance under our experimental protocol, with a screening accuracy of 98% (Baseline vs. cognitive-load conditions) and a staging accuracy of 87.32% (Mild vs. High load); (2) Feature importance analysis reveals that while total time serves as a universal biomarker across subjects, compensatory strategies under cognitive load are highly heterogeneous (e.g., systemic gait deterioration vs. conservative deceleration). These findings suggest that an adaptive, physics-guided approach is essential for robust home-based cognitive monitoring. By providing a low-cost, plug-and-play solution, this work paves the way for daily Parkinson’s disease risk screening in home-monitoring and resource-limited environments.

## Figures and Tables

**Table 1:** Information of experimental setups.

	Conference Room	Living Room
Height of Camera [m]	1.1	0.9
Far_Point [m]	4.2	4.6
Near_Point [m]	1.2	1.6

**Table 2:** Information of Subjects under test.

Subject Id	Height [m]	Age
S1	1.76	32
S2	1.78	28
S3	1.82	27
S4	1.68	24
S5	1.61	23
S6	1.85	33
S7	1.74	33
S8	1.71	22
S9	1.80	21
S10	1.78	22

**Table 4:** Evaluation on classification of health state.

	Precision	Recall	F1-Score
N	0.96	1.0	0.98
M	0.78	0.82	0.80
P	0.87	0.80	0.83

## Data Availability

The original contributions presented in this study are included in the article. Further inquiries can be directed to the corresponding author.

## Abbreviations

The following abbreviations are used in this manuscript:

TUG	Timed Up and Go
CMI	Cognitive-Motor Interference
MEMS	Micro-Electro-Mechanical Systems
PD	Parkinson’s Disease
ST	Single-Task
DT	Dual-Task
LASSO	Least Absolute Shrinkage and Selection Operator
LOOCV	Leave-one-out cross-validation
FOV	Field of view
STS	Sit-To-Stand
STS2	Stand-To-Sit
